# Auto-Intoxication

**Published:** 1914-10

**Authors:** J. W. M’Laughlin

**Affiliations:** Austin, Texas


THE
### TEXAS MEDICAL JOURNAL
Dr. F. E. Daniel, Founder. Established July, 1885
MRS. F. E. DANIEL, - Publisher and Managing Editor
Published Monthly.—Subscription, $1.00 a Year.
VOL. XXX AUSTIN, OCTOBER, 1914 No. 4
The publisher is not responsible for views of contributors

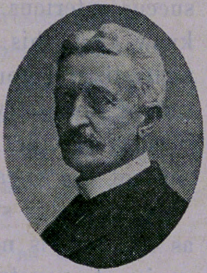

### Original Articles.

For Texas Medical Journal.

### Auto-Intoxication.

BY J. W. M'LAUGHLIN, M. D., AUSTIN, TEXAS.

In presenting to you the subject of auto-intoxication, I fully realize that I am attempting to cover a field that is as yet undeveloped, and, in consequence, many theories as to its cause have been presented, and nearly equally as many have exploded, with the result that we are very little nearer the goal than when it was first noticed.

This paper, therefore, is not a discovery, nor have I theories of my own to advance, but I am merely attempting to discuss and report for your consideration a few selected cases that have come under my observation.

The nitrogenous foodstuffs furnish the most toxic substances, particularly the ptomaines, and they are the most potent factors in causing auto-intoxication; that is, as far as the foodstuffs are concerned; so, therefore, where there is a diseased condition in the stomach, either secretory or motor, causing faulty proteid conversion, we can easily see how much more magnified the putrefactive changes will be in the colon; as, for instance, hydrochloric acid and pepsin should be secreted in their proper proportions, but, where there is a marked increase of hydrochloric acid, the pepsin is destroyed, and, as the two are not in proper proportions, the result is that the proteids pass to the duodenum unchanged. Being in this condition, it is of course harder for the pancreatic secretion to convert them into the absorbable foodstuffs or peptones. Again, with this enormous output of free hydrochloric acid by the stomach, it requires the gall bladder, pancreatic secretion and succus entericus to also increase their amounts of secretion, as we know that this acid bolus coming from the stomach must be neutralized befor pancreatic digestion will take place. As a result of this overwork by the gall bladder and pancreas, we get an insufficiency, or, better still, a sluggish condition, hence the symptoms of the so-called bilious attacks and auto-intoxication.

It is in this stage of the disease that complications may arise, as the field is now ripe for the colon bacillus or some other organism to penetrate deeper into the system, thereby producing an infection of the gall bladder—hepato-intestinal toxemia.

It is in this stage also that we frequently get a duodenitis; whether due to the colon bacillus infection, or due to the action of the acid bolus coming from the stomach and acting as a direct irritant, is a question that I am unable to say; but, nevertheless, the condition goes to increase the auto-intoxication symptoms.

These foodstuffs in an unprepared state reach the duodenum, and the pancreatic secretions convert what they can, and the balance pass on to the colon, where it is immediately attacked by the flora of bacteria, there producing a colitis and undergoing putrefactive changes with the production of indols, etc.

So far, our discussion has been of those conditions leading to the production of auto-intoxication, resulting from the hyperacidities, and we can readily see that in a hypo or low acidity of the stomach, where there is a deficiency of hydrochloric acid secretion, that our nitrogenous foodstuffs suffer in like manner, only we do not get the duodenal irritation from the acid, but the field is always open for an infection.

In a myasthenia gastrica and intestinalis, due to the loss of motor tone of the gastro-intestinal tract, this sluggish condition predisposes towards fermentation and putrefaction, hence we get an auto-intoxication.

Frequently we see cases where the cecum is widely dilated; so much so that at times it extends over to the mid-abdominal line, and it is in these cases of dilated cecum, or typhlatonia, that we get the most profound auto-intoxication symptoms, and it is through this media that frequently we get systemic infections of the colon bacillus, as this region under ordinary circumstances is rich in bacteria, and under pathological conditions it is doubly so.

Allowing this condition to run on, we soon get an incompetent ileo-cecal valve, with a result that the cecal contents regurgitates in the ileum, thereby producing an iliac stasis, characterized by more profound toxemia and dull pain in the lower abdomen. It is in this condition that the internest is especially interested as it is he who will be expected to give relief, as all surgical means, such as plication, suspension, etc., have thus far failed.

I want to say a few words in regard to the toxemias produced by the colon bacillus, as I believe that we have lots to learn as to what part this organism plays in the production of auto-intoxication.

Normally in the colon we have the colon bacillus as well as many others, and under ordinary conditions they seem to produce no ill effects, but under pathological conditions they become more virulent, as the following cases will show:

Case No. 1.—Mrs. P., aged 26; female; occupation, housewife. Examined August 29, 1913, and gave the following history:

Twelve weeks ago the patient was taken with a chill, followed by a fever and pains in the joints and bones. No history of any previous trouble of such a nature; has had no children, no miscarriages, no malaria, no typhoid, no pneumonia, but has always been very nervous.

Present Condition: No appetite, no vomiting, but feels sick and dizzy and pain upon pressure in the right hypochondrium of a dull nature; also very severe pain in the larger joints, more especially wrists and knees.

Inspection: Patient thin, anemic and cyanosed, eyes jaundiced, larger joints swollen and red. Temperature ranging from 99 to 101.

Physical Examination: Pain upon pressure over gall bladder, that is not affected one way or the other by meals. Cecum dilated and splash sounds present. Heart, lungs and other organs normal.

Microscopical Examination of Blood: Polynuclears slightly increased; cells pale, and malaria negative.

Urine Examination: Indican excessive, otherwise normal.

On August 11th patient's left arm was thoroughly sterilized and several c.c. of blood was drawn directly from the median vein and placed in sterile vacuum bulb, so as to eliminate all chances of contamination.

Result: The bouillon culture (100 c.c. beef extract bouillon containing 2 c.c. of patient's blood).

After eighteen hours' incubation it contained an even clouding, with slight pellicle formation. Stained smears from this culture revealed a gram negative, short thick bacillus. Hanging drop showed actively motile bacilli, whose motility was apparently the same as the colon bacillus and not so actively motile as the bacillus typhosis. Plate cultures developed in twelve hours, small, porcelain, round, glistening dense colonies, with continuous edges. In double sugar (Russell's media) tubes, these bacilli showed acid above and below the abundance of gas.

We did not have on hand any immune serum, so that agglutination test could not be applied, but cultures were sent to Johns Hopkins and the army, with result that both pronounced the bacillus as colon.

A second culture was made September 4, 1913, with exactly the same result as before.

On September 5th I gave the patient an autogenous vaccine, prepared from the culture containing fifty million bacilli, which caused no reaction other than a slight increase of pain in joints affected and a slight lowering of temperature.

On September 10th I gave the second injection of one hundred million and on September 14th I gave the last injection of two hundred and fifty million bacilli, with the result that the patient's rheumatism, pain over the liver, jaundice and all auto-intoxication symptoms disappeared, and she felt entirely well; better than she had in years.

Case No. 2.—Mr. G., aged 35; married; occupation, merchant.

Family History: Father died of tuberculosis.

Present History: Has had no sickness with the exception of rheumatism, and this commenced in 1911. Drinks one cup of coffee a day, several glasses of beer and many cigarettes; masticates his food well, but has gastric distress immediately and six hours after meals; dizzy, nervous, drowsy in the mornings, and has abdominal discomfort all the time, so much so that a belt or anything tight around the waist-line is very uncomfortable. Patient has had very severe attacks of rheumatism, and as a matter of fact he has never been free from pain since 1911.

Physical Examination: Liver slightly enlarged, very tender over gall bladder and duodenum. Head zones for gall bladder irritation very positive.

Inspection: Eyes jaundiced, otherwise normal.

As we could get no culture from the blood, we sterilized a vessel and obtained a bowel action. From this we made growths of colon bacillus, and from this we prepared our autogenous vaccine.

On April 22, 1914, I gave him his first injection of three hundred million bacilli, and every five days thereafter I gave him an injection, each time increasing three hundred million until two billion was reached.

Patient is now free of all symptoms; jaundice has entirely disappeared, and he is in every way normal.

Case No. 3.—Physician, aged 45. Up until two years ago the doctor had always enjoyed good health, and dates his present trouble back to a night when he, being very tired, had to eat hurriedly and rush to a maternity case, which proved to be difficult and tedious. As the patient was not properly prepared, there were frequent evacuations from the bowel, and the doctor had great difficulty in keeping his hands and the patient clean.

After having left this case he noticed his face burning, and recalls rubbing his hand over it frequently. A few days later there developed quite a rash, which extended over his face and hands. This he took to be a seborrhea, which diagnosis was verified afterwards by several others.

Possibly two weeks after the rash had made its appearance, he began to notice gastro-intestinal symptoms, such as dull feeling all over, metallic taste in mouth, poor appetite, feeling of fullness and weight in abdomen, more marked after meals, but which persisted more or less all the time. The bowel acted regular each morning, but occasionally there developed a diarrhea that could not be accounted for, as he was very careful in regard to his diet.

On May 26, 1914, I examined the doctor, and found the following pathological conditions: A total absence of free hydrochloric acid, with a combined acidity of 14 and a total acidity of 22; lactic acid was present, and microscopically the stomach contents contained numerous colon bacilli.

The urine was normal other than a marked excess of indican.

As it was impossible for the doctor to remain in Austin, I prescribed gastric lavage three times a week, colon irrigation twice a week and an autogenous vaccine prepared from the bowel action every four days.

I might say that we failed to get a culture from the seborrhea where the scrapings were taken from the face.

On June 2d he reported for his second examination, and I found that the seborrhea had nearly entirely disappeared; that his gastro-intestinal symptoms had improved, but upon clinical examination of the stomach contents there was no change other than there were no colon bacillus present.

Case No. 4.—Mrs. R., aged 47; married. No sickness since childhood, other than gastro-intestinal symptoms, which have persisted for several years, manifesting itself by vertigo, bad taste in the mouth, fullness and gas in abdomen, accompanied by a dull pain in the right iliac fossa.

Upon physical examination, I found a dilated cecum, accompanied by a marked iliac stasis.

Clinically, the stomach showed no secretory disturbance, but all evidences of a motor insufficiency. The urine contained an enormous amount of indican.

For this patient, I used the abdominal straps, as the abdomen was very flabby; prescribed duodenal irrigation, consisting of two quarts of normal salt solution at 110° F. each morning for two weeks and a tonic of I. Q. S.

After two weeks' treatment the cecum had contracted back to normal, the patient had gained several pounds in weight, all symptoms had disappeared, and as far as I could see there was no further evidence of an auto-intoxication.

